# Genetic parameters and pedigree–based selective breeding outcomes for productivity traits in industrial black soldier fly (*hermetia illucens* L.) production systems

**DOI:** 10.1186/s12711-026-01088-z

**Published:** 2026-09-16

**Authors:** Armel S. L. Donkpegan, Alexandra Guigue, Nicolas Berteau, Léo Compernolle, Romuald Rouger

**Affiliations:** 1 SYSAAF, UMR BOA, Centre INRAE Val de Loire, Nouzilly, 37380 France; 2 INNOVAFEED, 85 rue de Maubeuge, Paris, 75010 France

## Abstract

**Background:**

Selective breeding in mass-reared insects has progressed rapidly in recent years, and the black soldier fly (*Hermetia illucens* L. - Diptera: Stratiomyidae), the most widely cultivated insect species for animal feed, is emerging as a strong candidate for genetic improvement. Estimation of quantitative‑genetic parameters and implementation of pedigree‑based selection schemes are essential to increase genetic gain while controlling inbreeding under industrial rearing. Here, we report a pilot‑scale breeding experiment conducted under family group‑mating with partial pedigree uncertainty, designed to assess the genetic parameters of key growth and reproductive traits in *H. illucens* and to evaluate the feasibility of pedigree‑informed selection in an industrial context. Genetic parameters were estimated using multivariate REML animal models and breeding values were predicted using Best Linear Unbiased Prediction. The feasibility of systematic genetic improvement under industrial rearing conditions was assessed through seven generations of family selection.

**Results:**

A total of 15 179 individuals from 266 full-sib isofamilies were phenotyped. Heritability estimates (± SE) were: larval weight at stage 1 (LW1, h^2^ = 0.12 ± 0.08), stage 2 (LW2, h^2^ = 0.13 ± 0.04), restricted-feed LW2 (LW2R, h^2^ = 0.58 ± 0.03), female body weight at emergence (FEMBW, h^2^ = 0.006 ± 0.005), pupal perimeter (PERIM, h^2^ = 0.06 ± 0.09), oocyte number (OOCY, h^2^ = 0.43 ± 0.09), and thorax size (THOR, h^2^ = 0.40 ± 0.09). Several traits exhibited substantial environmental variance. Larval body-weight traits (LW1, LW2, LW2R) exhibited positive genetic correlations with thorax size and oocyte number, while female body weight at emergence was negatively correlated with larval body weight. In this pilot‑scale programme, seven generations of selection for larval body weight yielded indicative genetic improvement, tempered by design constraints and a detectable risk of inbreeding.

**Conclusions:**

These multi-trait genetic parameter estimates indicate that several economically important traits in*H. illucens*are moderately to highly heritables. Although genetic correlations were generally trait-dependent and could be either positive or negative, supporting the potential for genetic improvement through appropriately designed multi-trait breeding strategies. Patterns observed for larval body weight are indicative of a genetic response under industrial conditions, although environmental contributions cannot be fully excluded in the absence of a control line. Together, these results suggest the potential for genetic improvement in*H. illucens*populations, provided that inbreeding is carefully managed through adequate selection nucleus size and pedigree organisation.

## Background

The insect farming sector is expanding rapidly in response to the growing demand for sustainable protein sources. Production forecasts for edible-insect products indicate substantial industrial development in the coming years, with estimates reaching approximately 260,000 tons by 2030 across the globe [1]. Among the species currently mass-reared, *Hermetia illucens* Linnaeus, 1758 (Diptera: Stratiomyidae), commonly known as the black soldier fly (BSF), has emerged as one of the most promising candidates for large-scale feed production. This saprophagous species exhibits high bioconversion efficiency and the ability to up-cycle a wide range of organic side-streams [2–4]. Owing to their favourable nutrient profile, *H. illucens* larvae constitute a high-quality feed ingredient for livestock, poultry, aquaculture species, and other animal production systems [5–7].

Selective breeding of *H. illucens* has recently become a central focus of the insect‑production sector, as industrial competitiveness increasingly depends on achieving faster growth and improved biological performance [8–10]. However, developing effective breeding programs in this species remains challenging due to its short life cycle, limited knowledge of its reproductive biology, and the practical constraints of managing large cohorts under industrial conditions. These challenges highlight the need for robust methodologies and protocols specifically adapted to *H. illucens* breeding. Recent studies have demonstrated substantial genetic diversity within *H. illucens* populations, indicating that selective breeding has strong potential to generate meaningful genetic progress [11, 12]. This evidence has stimulated growing interest in improving traits of economic relevance under controlled rearing conditions. Consequently, the first research efforts have addressed early domestication dynamics [13] and response to selection for key phenotypic traits [14], providing an initial framework for structured genetic improvement programs in this species.

Several recent studies have reported estimates of quantitative genetic parameters in *H. illucens*, reflecting the growing recognition that such information is essential for development of effective breeding programs. Since the first investigation estimating heritabilities for traits of economic interest using linear mixed models in this species [15], additional studies have been published over the past two years [16–18]. Reported heritability estimates include 0.25 for prepupal body weight, 0.45 for pupal body weight, and 0.38 for development time to the prepupal stage, although these estimates were associated with relatively large standard errors [15], underscoring the need for more comprehensive datasets and improved pedigree structures to refine genetic parameter estimates in this species.

A study using a full‑sib design to estimate the heritability of larval and prepupal body sizes reported notably inflated estimates, reaching 0.67 and 0.78, respectively [17]. More recently, an unconventional approach for estimating genetic parameters based on SNP variation and, therefore, not directly comparable to pedigree‑based estimates, demonstrated that SNP‑based heritability was significant for growth traits but low and non‑significant for fitness‑related traits [16]. Finally, a more comprehensive investigation recently provided the first estimates of genetic parameters for larval size assessed through individual larval surface area (ISA), group surface area (GSA), and group weight (GW), using a REML‑based mixed model framework [18]. These results highlight the significant genetic contribution to the expression of these traits, which can be harnessed to improve *H. illucens* strains for industrial applications.

Building on the relatively high heritability estimates reported for body‑weight traits in *H. illucens*, several artificial selection programs have recently been initiated [14, 19]. The first long‑term selection study implemented a breeding program over 16 generations, comparing the performance of a line selected for increased larval body weight with that of an unselected base population across six experimental trials conducted under different rearing conditions. Under automated production settings, the selected line showed substantial improvements relative to the base population, including average increases of +39% in larval weight, +34% in wet crate yield, +26% in dry‑matter yield, +32% in crude protein yield, and +21% in crude fat yield [14]. These results illustrate the strong potential for genetic improvement of production traits in *H. illucens* under controlled industrial conditions. Second, the long‑term consequences of selection for increased body weight on reproductive performance in *H. illucens* have been assessed by comparing egg clutches produced by a line selected for larval body weight over 14, 21, and 32 generations with those of an unselected base population. Across four years of evaluation (2021–2024), the selected line exhibited consistently superior reproductive output, with increases of 18 to 49% in clutch weight per female, 24 to 30% in the number of eggs per clutch, and 3 to 4% in egg length relative to the unselected base population [19]. Importantly, both studies were based primarily on mass selection schemes. While such approaches have demonstrated considerable potential for improving productivity in *H. illucens*, their capacity to manage inbreeding and optimise long-term genetic gain remains limited. These limitations highlight the potential value of pedigree-based breeding strategies for supporting sustainable genetic improvement in industrial black soldier fly production systems.

Pedigree‑based methods rely on documented genealogies to estimate relationships among individuals and have been widely and successfully applied in livestock and aquaculture species to increase selection accuracy and genetic gain [20, 21]. Selection using estimated breeding values (EBVs) specifically targets the heritable component of phenotypic variation, particularly the additive genetic variance, thereby providing more accurate predictions of offspring performance. Compared with phenotypic selection, EBV‑based selection reduces sensitivity to local environmental effects because phenotypes are corrected for systematic environmental variation and relationships among individuals are explicitly accounted for [22].

An increasing number of studies in insects have incorporated pedigree information to estimate genetic parameters and predict breeding values [23–26]. These works, conducted in species such as the housefly, black dump fly, and mealworm, demonstrate the feasibility of applying animal-model analyses when individual pedigrees can be recorded reliably within small groups. The species that these studies were conducted in exhibit social and mating behaviours that facilitate controlled pairings or low-density group matings, enabling accurate reconstruction of genealogies. In contrast, implementing comparable pedigree-based approaches in more gregarious insects such as *H. illucens* is considerably more challenging because of the species’ intrinsically group‑dependent reproductive system, which renders solitary mating largely ineffective. Instead, males engage in competitive mating behaviour and large groups are required to ensure adequate mating success. These biological and behavioural features limit the feasibility of obtaining individual parentage information and complicate the design of classical pedigree-based breeding schemes. Previous research consistently indicates that successful copulation occurs only under conditions where conspecific aggregation is present, highlighting the essential role of social context in enabling reproductive success. [27–29]. While this may indeed remain true under certain natural or semi-natural conditions, recent studies have reported contrasting evidence, demonstrating that individual mating events between isolated males and females can occur [18, 24].

The aim of this study was to develop a full-sib pedigree-based breeding scheme for *H. illucens* that enables individual-level phenotyping and estimation of genetic parameters for seven traits of economic relevance. Within this framework, we evaluated the expected genetic progress achievable through family-based selection and assessed the applicability of these methods to large-scale industrial production systems.

## Methods

### Experimental setup of the *H. illucens* breeding population

#### Egg collection and early development protocol

The nucleus population of our experimental line was created by using an hundred adult population of *H. illucens* sourced from INNOVAFEED (2 rue de l’Europe, 59,231 Gouzeaucourt – FRANCE), with a sex ratio close to 1:1. Two days following the confinement of adult specimens in mating cages, oviposition was monitored and collected over a period of 1 to 2 days. Egg deposition was facilitated using custom corrugated cardboard oviposition collectors, which were positioned atop a specialized oviposition substrate that consisted of wheat bran and vinasse, preconditioned for one week at 30 °C to enhance its attractiveness and stimulate egg-laying behaviour. The collectors were handcrafted and designed to enable egg collection by well and on a per‑female basis. To reduce the probability of multiple females laying on the same structure, the cage contained several independent oviposition sites. Eggs were never collected at the group level: individual clutches deposited on distinct sites and separated by physical discontinuities on the substrate were isolated, collected independently, and treated as candidate single female clutches. To ensure that each collected egg batch originated from a single female, we first weighed clutches produced by individually isolated females immediately after mating, in order to establish a reference interval for the mass of a single *H. illucens* clutch. All egg batches whose measured mass exceeded the boundaries of this reference interval were excluded.

Thus, each egg collection obtained from a single female was referred to as an isofamily and was defined as a group of siblings originating from a single clutch (from eggs to adults, including larvae). We assumed that this production resulted from a single male–female mating. Each egg clutch, corresponding to the oviposition of a single female, was individually harvested and transferred onto a plastic cap, following the protocol described in [30]. From this point onward, each clutch was uniquely labelled with its generation identifier, the parental cross (cage ID), and a clutch number. Prior to hatching, the individual mass of each clutch was recorded using a precision balance. The plastic cap containing the clutch was then placed into a container containing 30 g of substrate with 70% humidity, which serves as a buffering medium to support the immediate post-hatch development of juveniles. Upon hatching, each clutch was reweighed to estimate the hatching success rate and quantify the number of juveniles present within the substrate. Based on this estimate, the appropriate feed ration was calculated for the pre-growth phase, which spans from day 0 to day 7 post-hatching.

#### Juvenile transfer and pre-growth phase

The buffer substrate containing the newly hatched juveniles was transferred into larger containers, each designated for the development of a specific isofamily. The feed ration required for the pre-growth phase was approximately 0.192 g of fresh matter per larva, and was adjusted based on the estimated number of juveniles per clutch. The pre-growth substrate was formulated from wheat bran, wheat vinasse, and water, with a target moisture content of approximately 70%. Environmental conditions during this 7-day pre-growth phase were maintained at 27 °C (±3°C) and 70% relative humidity. At the end of the juvenile emergence phase, larvae were separated from their frass and the consumed pre-growth substrate using a sieving process. The larvae were then inoculated into a growth substrate with a moisture content of 72%. To estimate survival rates from day 0 to day 7, a subsample from each isofamily was weighed to approximate the number of surviving juveniles. For standardization, 500 larvae were allocated per growth container to ensure uniform density across isofamilies. The feed ration for the growth phase was set at 1.7 g of fresh matter per larva, totalling 850 g per container, which supports larval development from day 7 until pupation. This rationing protocol aligns with that used for larvae destined for reproductive purposes (“mating”). During the growth phase, environmental conditions were maintained at 30 °C and 50% relative humidity. Each isofamily developped from the larval to adult stage within plastic containers measuring 11 cm in diameter and 12 cm in height.

#### Sexing, adult rearing, controlled mating procedure, and pedigree reconstruction

Adults from each selected isofamily were sexed and placed into mating cages measuring 30 × 30 × 30 cm. Each cage contains 40 individuals, composed of 20 males from one isofamily and 20 females from a different isofamily, with each cross uniquely identified. All possible combinations were performed when sufficient numbers of individuals are available. To prevent sibling mating within the same isofamily, only adults that were less than 24 hours post-emergence were used for crossing. The mating cages were then placed inside a controlled environment tent maintained at 27 °C (±3°C) and 70% relative humidity. The lighting system consists of UV LED bulbs (365 nm), green light (515 nm), and blue light (450 nm). The cages were positioned 20 cm below the lighting ramps, each of which contains 4 blue bulbs, 4 green bulbs, and 6 UV bulbs, illuminating 4 mating cages simultaneously. The adult rearing phase lasts on average 4 to 5 days.

**Fig. 1:**
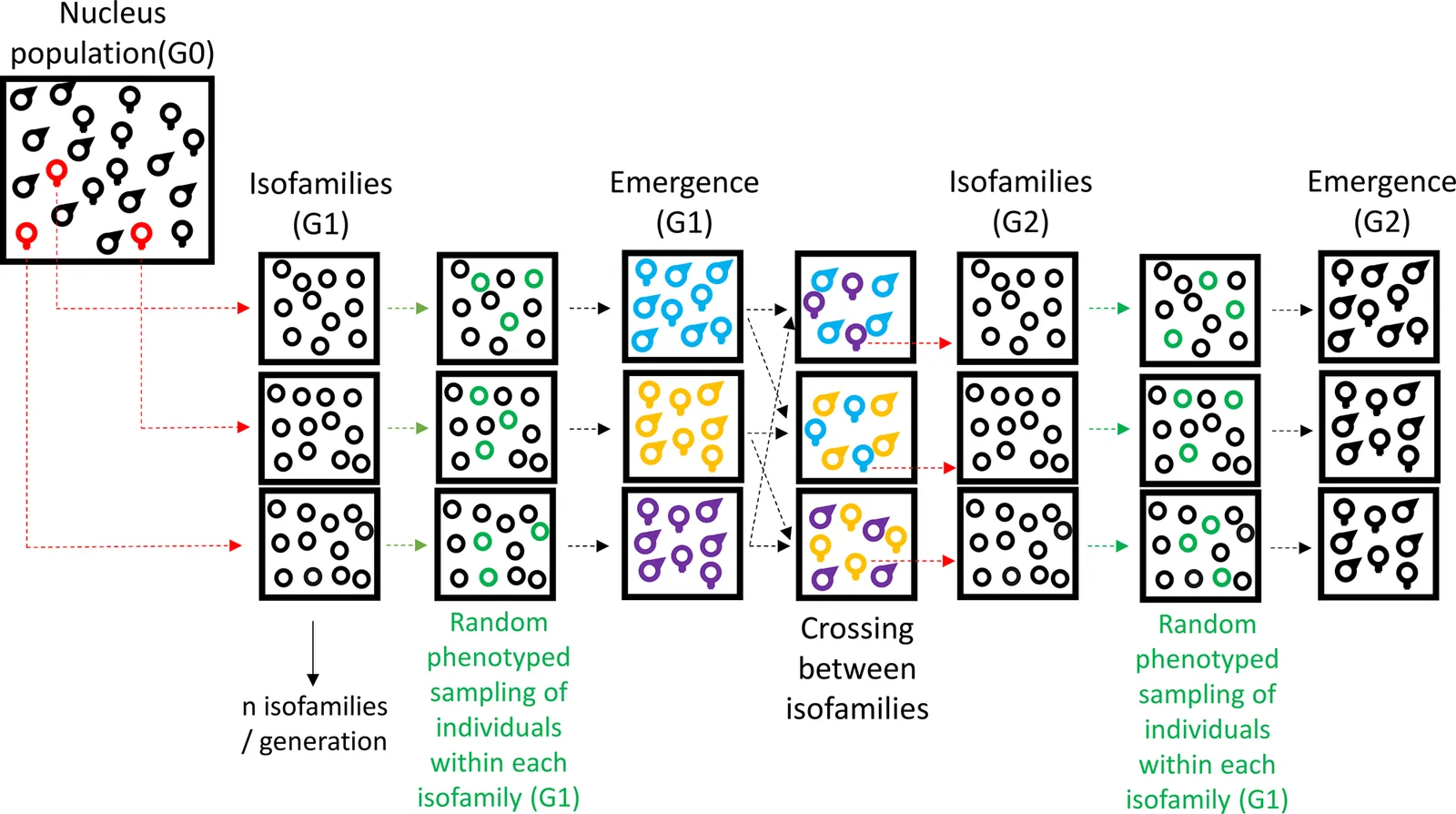
Schematic representation of the family-based pedigree selection program developed for *H. illucens*. Generation G0 consists of larval populations originating from multiple sources (production stocks and R&D colonies). Isofamilies are established from egg batches collected from individual G0 females, with each batch corresponding to one isofamily. After incubation and early larval development, a subset of larvae from each isofamily is randomly sampled and phenotyped. These phenotypic data, combined with recorded pedigree information, are used to estimate breeding values that guide the identification of the top-performing isofamilies. The selected isofamilies are then intercrossed with males from one isofamily mated with females from another to generate the next generation. The process is repeated each cycle to achieve cumulative genetic improvement

Considering that adults mated freely within each 20 × 20 cage and no individual parentage was tracked, we did not attempt to assign specific sire–dam pairs to each clutch. Instead, we used a pseudo‑pedigree structure in which: (1) Each mating cage was treated as a single parental group; (2) All males in the cage were considered as potential sires and all females as potential dams; (3) Each clutch was therefore linked to a parental group identifier, rather than to a specific male and female. This approach corresponds to the standard “group mating pedigree” structure used in mass‑selection or isofamily‑based breeding programs under multiple mating, in which individuals were treated as maternal half‑sib groups with shared paternal contributions. Building successive generations, several clutches were collected per cage to maintain the required number of families per generation. These clutches were assumed to be full‑ and half‑sib families. Multiple clutches from the same cage were treated as separate maternal isofamilies with shared parental group and the pedigree links each clutch to the same parental group rather than to a unique parental pair. Figure 1 illustrates a schematic representation of the family pedigree selection program.

#### Phenotypic measurements

Within each full and half-siblings isofamily produced per generation, the first phenotype (LW1) was recorded a few days after hatching, using a derived batch of larvae, which were then destroyed. A few days later, the second set of phenotypes (LW2 & LW2R) was recorded using another derived batch of larvae. And finally, all other phenotypes were measured just after pupal emergence (PERIM, FEMBW, OOCY & THOR), using several derived batches of pupae.

The description of all phenotyped traits measured were:

Larval Weight 1 (LW1): At the end of the first growth phase (i.e. 7 days after hatching), the containers were sieved and 30 random larvae per isofamily were sampled. Larvae were then frozen and stored at −20 °C. Larvae were then thawed, gently wiped and weighed on a precision scale (Radwag AS 220.r2; 0.1 mg). The remaining larvae were fed to start the second growth phase (1,7 g of feed/larvae).

Larval Weight 2 (LW2): 7 days after the start of the second growth phase (i.e. 14 days after hatching), 30 random larvae per isofamily were sampled. Weighing protocol was the same than for LW1.

Larval Weight 2 Ratio (LW2R): Starting at generation 11, one third of the larvae per family (around 170 out of 500) were provided with a limited feed ratio (0, 75 g/larvae) from the end of the first growth phase onward. Under these limiting conditions, 30 individuals per isofamily were individually weighed at the end of the second growth phase.

Pupal Perimiter (PERIM): When an isofamily reaches a pupation rate of at least 75%, 30 pupae were randomly sampled and positioned on a 10-millimeter translucent polycarbonate plate, engraved with 20 × 4 cells (internal: 26 × 6.5×4 mm), used to optimise the position of the pupae and prevent overlapping. The plate was placed on a white A3 LED drawing board. A 4K camera (Brio Logitech Capture) placed above films the plate. Using a custom-made OpenCV 3 Python Script, the pupal perimeter is measured in pixels and converted in millimetres. The pupae were then put back in their containers until emergence.

Oocyte (OOCY) and Thorax (THOR): Upon emergence, more than 10 females were randomly sampled from each isofamily, isolated in reproduction cages and provided with water until they reached three days of age. Flies that died before three days were discarded, and the remaining ones were frozen and stored at −20 °C until dissection. Dissections were performed in Petri Dishes filled with water. Each fly was secured with a dissection pin. The abdomens were carefully incised with a scalpel and both ovaries were removed. One ovary was put on a glass slide, a drop of paraffine was added, and the ovary was gently pressed on the slide with a glass coverslip until all the oocytes formed a unique layer. At this age, most ovaries still had round oocytes, and the coloration varied between translucent to completely white. A picture of each ovary was taken by a 5.1 mpx camera (ODC 825, Kern and Sohn GmbH) under a trinocular stereomicroscope (OZM 544, Kern and Sohn GmbH). Oocytes were then manually counted using Microscope VIS basic software. Preliminary work indicated no significant difference in oocyte numbers between left and right ovaries (unpublished). Only one ovary per female was therefore counted.

The thorax was separated from the abdomen and the head, and all remaining appendages were removed. The thorax size was measured between the extremities of the pronotum and the scutellum using the same set-up, with a calibration slide, as for the oocyte counting.Female Body Weight at emergence (FEMBW): Isofamilies were checked every 24 hours for emergence (except on weekends). Shortly after the emergence peak, where individuals were kept as reproducers for the next generation, females were collected shortly after emergence (blue coloration of the abdominal window, greyish cuticle), frozen, and stored at −20 °C, before they were weighed on a precision scale (Radwag AS 220.r2; 0.1 mg).

All data processed will be recorded in the pedigree and the remaining non-phenotyped individuals are used for breeding after selection (LW2 case only).

To avoid any ambiguity between phenotyped and breeding individuals, we explicitly note that phenotyping was always performed on a predefined subset of individuals within each family, and that none of these phenotyped individuals contributed genetically to the next generation. For larval traits, on average 30 individuals were sampled per isofamily and immediately frozen for phenotypic measurements. All remaining siblings from the same family were left undisturbed and continued development to adulthood. For adult traits, a small subset of emerging females was phenotyped for each trait, and these individuals were also excluded from breeding.

### Genetic parameters and breeding value estimation

Although the number of phenotyped individuals fluctuated depending on the trait of interest, phenotypic variability was assessed for each recorded trait. All generations were evaluated under the same controlled conditions. Phenotypic analyses were performed using R 4.0.3 software. [31]. Estimation of (co)variance components, followed by calculation of genetic parameters, was conducted using seven-trait animal model by the REstricted Maximum Likelihood method (REML), using VCE 4.0 programs [32, 33]. For estimation of variance-covariance components and genetic parameters, the following model was used in Eq. (1):

1 $${\bf{Y}} = {\bf{X}}\beta + {\bf{Za}} + {\bf{Wf}} + {\bf{e}},$$

where **Y** is the vector of observations for the investigated traits (see Table 2 for details); **β** is the vector of fixed generation effects (2 to 13 levels depending on the analysed traits - see Table 2, column 9 for details); **a** is a vector of random genetic additive; **f** is a vector of random isofamily effects (i.e. common environmental effect of sib-group growing in the same batch); and **e** is a vector of random residual effects. **X**, **Z** and **W** are the incidence matrices related to these effects. Variance matrices of random effects are as follow:

2 $$V\left({\bf{a}} \right) = {\bf{A}}{\sigma _a}^2,$$

3 $$V\left({\bf{f}} \right) = {\bf{I}}{\sigma _f}^2,$$

4 $$V\left({\bf{e}} \right) = {\bf{I}}{\sigma _e}^2,$$

where **A** is the numerator relationship matrix calculated based on the pedigree and I is an identity matrix. Additive genetic variance contribution was quantified by heritability (h^2^), computed as in Eq. (5):

5 $${h^2} = {\sigma _a}^2/({\sigma _a}^2 + {\sigma _f}^2 + {\sigma _e}^2),$$

and contribution of the common environmental effect was estimated by Eq. (6):

6 $${c^2} = {\sigma _f}^2/({\sigma _a}^2 + {\sigma _f}^2 + {\sigma _e}^2),$$

Genetic and phenotypic correlations were calculated from the (co)variance estimates of the multi-trait animal models; and the R package ‘PEDIGREE’ [34] was used to calculate the inbreeding levels of each individual at each generation.

Genetic evaluations for genetic gain prediction for LW2 were obtained from the Best Linear Unbiased Predictor (BLUP) implemented in the BLUPF90 software packages procedure [35], which was used to estimate breeding values (EBVs) for all animals from G4 to G12. The same models as for genetic parameters estimation were used for breeding value estimation, but using univariate models, as selection was on a single trait (LW2). At this stage, phenotyping was performed on a subset of on average 30 individuals per isofamily, sampled 14 days after hatching from derived larval batches. These sacrificed larvae provided all phenotypic records used to compute EBVs, which were averaged and assigned to each corresponding isofamily. The surviving full-sib siblings were retained and continued to serve as the potential breeders for the next generation. Following phenotyping, an additional 3 to 5 days were required to register the pedigree, prepare the datasets, compute EBVs, and rank isofamilies based on their average genetic merit. During this period, the breeding stock continued its development until adult emergence. Newly emerged adults were put to sleep using UV light a few hours before being transferred into mating cages. The adult rearing phase lasted on average 4 to 5 days. Importantly, only the non-phenotyped siblings from the selected isofamilies were used as breeders, ensuring that phenotypic information informed family-level selection decisions, while the genetic contribution to the following generation originated exclusively from the surviving, un-sampled individuals, thereby maintaining a strict separation between phenotyping and reproduction.

### Selection procedure for body weight LW2

No selection was implemented over the first 5 generations of the experiment (G0 to G4). Starting at G5, a selection pressure of 18 to 22% was applied to select the best isofamilies breeders based on their average EBV for LW2 in order to produce successive generations. This selection procedure was used until G12. Thus, 3/15, 3/17, 3/17, 6/27, 6/28, 6/28 and 5/23 isofamilies were selected for generations G5, G6, G7, G9, G10, G11 and G12. Furthermore, in G8, we experienced many oviposition problems and eggs from production (corresponding to G0) were collected and crossed with a limited number of collected clutches from this generation. For all selected generations, genetic gain levels were assessed in order to quantify the effect of selection on LW2.

## Results

### Descriptive statistics

**Fig. 2:**
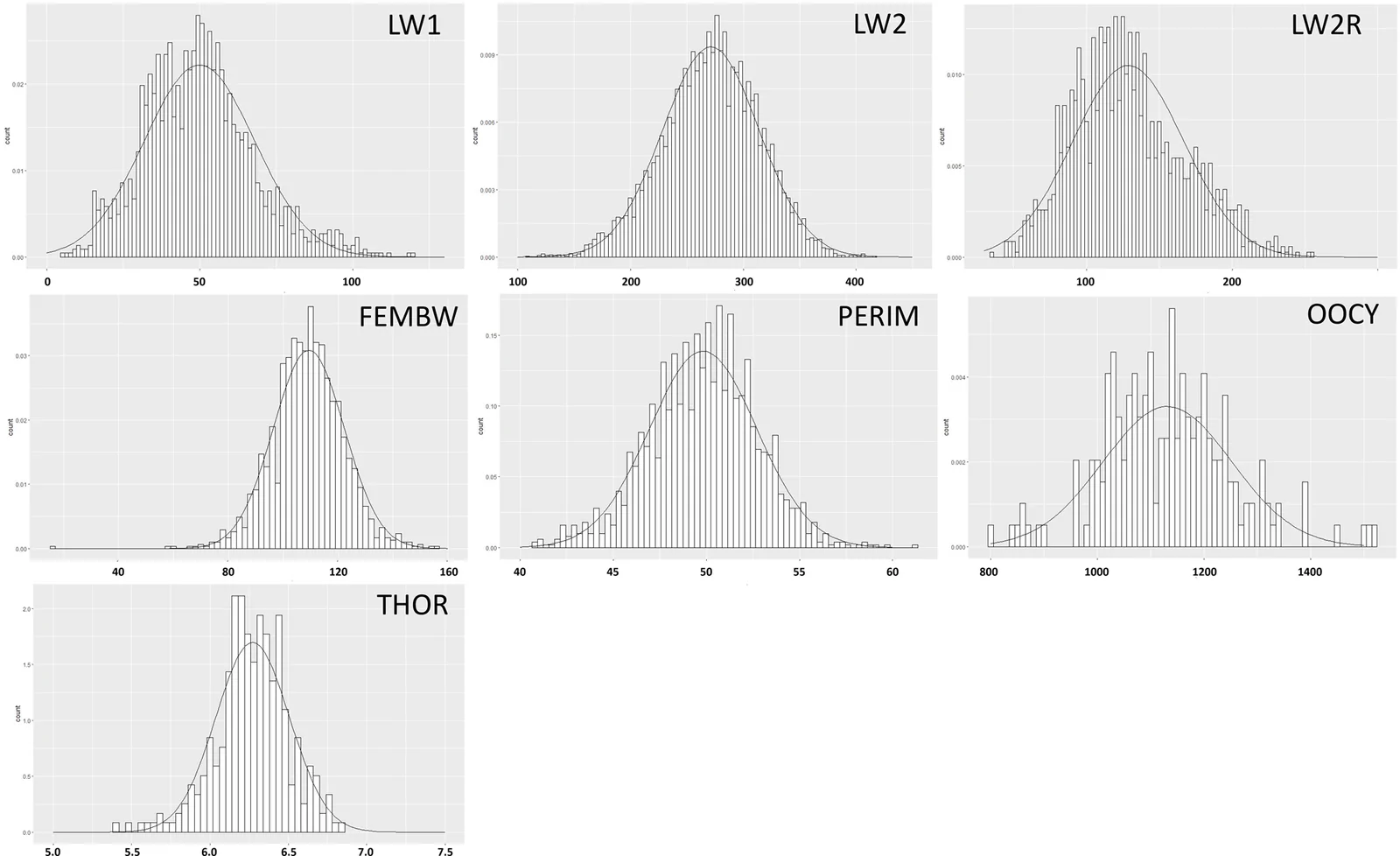
Individual and average phenotype distributions. All distribution panel are recorded at individual level for larval weight 1 (LW1, *n* = 1710), larval weight 2 (LW2, *n* = 8370), larval weight 2 Ratio (LW2R, *n* = 1398), female body weight at emergence (FEMBW, *n* = 1529), pupal perimeter (PERIM, *n* = 1680), number of oocyte (OOCY, *n* = 196) and thorax length (THOR, *n* = 296)

A total of 15,179 individuals from 266 full-sib isofamilies were phenotyped for estimation of genetic parameters and for the development of the experimental pedigree-based breeding program. All measured traits exhibited approximately normal distributions (Fig. 2). Summary statistics are provided in Table 1. Larval weight at stage 2 (LW2) was the most extensively recorded trait, with 8,370 phenotyped individuals, as it was the primary target of selection during the last seven of the thirteen generations evaluated.

### Genetic parameter estimates

To estimate genetic parameters, a total of 15,620 larvae were phenotyped and traced through the full-sib pedigree structure. Genetic parameters were obtained using BLUP under multivariate animal models. The estimates included heritability, environmental variance, and genetic correlations among traits, as presented in Tables 1 and 2. Heritability was very low for FEMBW and PERIM (0.006 and 0.06, respectively), whereas all other traits showed estimates of at least 0.10. Heritability for LW2 was markedly higher under restricted-feed conditions (0.58) than under standard conditions (0.13). As shown in Table 1, common environmental effects accounted for a substantial proportion of phenotypic variance for most traits, with c^2^ estimates ranging from 0.11 to 0.57. Only OOCY and PERIM displayed relatively weak environmental contributions (0.06 and 0.11, respectively).

**Table 1:** Summary statistics of traits of interest of *H. illucens* population

Traits	N	Mean	Vp	SD	Min	Max	CV (%)	nGen	**h**^**2**^*(SE)*	**c**^**2**^*(SE)*	**r**^**2**^*(SE)*	σ_*f*_^2^	σ_a_^2^	σ_e_^2^
LW1	1710	49.94	323.31	17.98	5	120.20	36.00	3	0.12 (0.08)	0.57 (0.04)	0.30 (0.05)	152.8 (24.2)	33.2 (22.3)	80.1 (10.3)
LW2	8370	271.15	1819.11	42.65	109	416	15.72	13	0.13 (0.04)*	0.15 (0.02)	0.72 (0.03)	237.5 (34.6)	207.0 (70.4)	1173 (37.9)
LW2R	1398	128.72	1448.17	38.05	34	254	29.56	2	0.58 (0.03)*	0.41 (0.04)	0.2e-8 (0.1e-4)	680.9 (103.6)	964.8 (32.0)	0.000 (0.019)
FEMBW	1529	109.33	167.18	12.93	16	156	11.82	2	0.006 (0.005)	0.25 (0.04)	0.74 (0.04)	42.95 (9.16)	1.05 (1.00)	127.6 (4.1)
PERIM	1680	49.83	8.27	2.88	40.67	61.10	5.77	3	0.06 (0.09)	0.11 (0.03)	0.84 (0.10)	0.86 (0.22)	0.46 (0.85)	6.81 (0.27)
OOCY	196	1131.49	14559.8	120.66	802	1516	10.66	3	0.43 (0.09)*	0.05 (0.03)	0.52 (0.10)	636.0 (343.3)	5619 (1864)	6727 (986)
THOR	296	6.27	0.05	0.23	5.38	6.83	3.74	3	0.40 (0.09)*	0.26 (0.06)	0.33 (0.08)	0.01 (0.004)	0.02 (0.01)	0.016 (0.003)

[Larval Weight 1 (LW1); Larval Weight 2 (LW2); Larval Weight 2 Ratio (LW2R); Pupal Perimeter (PERIM); number of Oocyte (OOCY); Thorax length (THOR); Female Body Weight at emergence (FEMBW)]. Number of samples size (N), Phenotypic variance (V_p_), Standard Deviation of Mean and Variance phenotype (SD), Minimum/maximum phenotype values (Min/Max), Coefficient of variation (CV), number of generations of data collected (nGen) Heritability (h^2^), proportion of phenotypic variance explained by common environmental effects (c^2^) of all traits estimated under BLUP model in *H. illucens* population, residual effect (r^2^). The presented variance components are the genetic variance (σ_a_^2^), common environmental variance (σ_*f*_^2^) and residual variance (σ_e_^2^)

**Table 2:** Estimates of genetic correlations (upper matrix) and associated standard errors (lower matrix) for traits of interest in a *H. illucens* population.)

Traits	LW1	LW2	LW2R	FEMBW	PERIM	OOCY	THOR
LW1		0.48	0.15	−0.33	−0.39	0.78	0.47
LW2	0.24		0.49	−0.91	0.39	0.43	0.87
LW2R	0.03	0.07		−0.13	0.71	0.27	0.30
FEMBW	0.12	0.20	0.20		−0.22	−0.25	−0.85
PERIM	0.46	0.41	0.66	1.39		−0.14	0.20
OOCY	0.16	0.27	0.12	0.55	0.89		0.11
THOR	0.05	0.07	0.05	0.17	0.64	0.23	

Larval Weight 1 (LW1); Larval Weight 2 (LW2); Larval Weight 2 Ratio (LW2R); Pupal Perimeter (PERIM); number of Oocyte (OOCY); Thorax length (THOR); Female Body Weight at emergence (FEMBW)

Estimates of genetic correlations are presented in Table 2. Because of large standard errors, most estimates could not be considered significantly different from zero. Nevertheless, strong positive genetic correlation estimates were observed among the three body-weight traits (LW1, LW2, LW2R), as well as between these traits and thorax size (THOR) and oocyte number (OOCY). In contrast, female body weight at emergence (FEMBW) showed unexpected negative genetic correlation estimates with the larval body-weight traits (LW1, LW2, LW2R).

### Selective breeding for LW2 and inbreeding dynamics in the experimental line

**Fig. 3:**
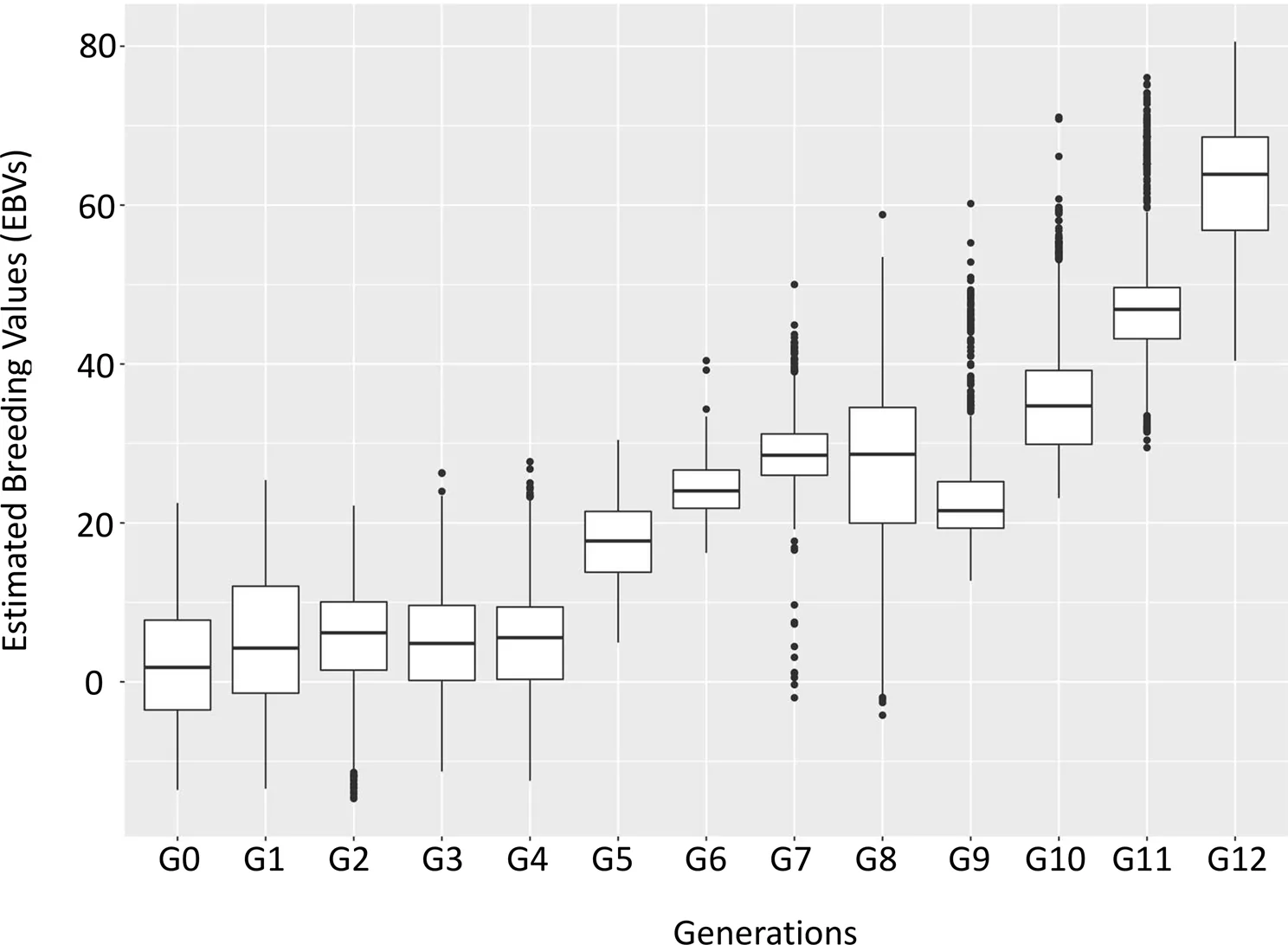
Evolution of the variance of estimated breeding values through 13 generations of selection for larval body weight (LW2) under the pedigree-based breeding program in *H. illucens*

During the first five generations (G0–G4), no selection was applied. Isofamilies were randomly crossed to produce successive generations, and the mean EBV for LW2 remained essentially stable across this period (Fig. 3). Selection began at generation G4, with an average selection intensity of approximately 20%. Over the next three generations, genetic gain initially declined (G4–G5: +248.6%; G5–G6: +38.5%; G6–G7: +18.8%), followed by a slight negative reduction at G8 (−2.6%) and a more pronounced drop at G9 (−13.8%). At G8, substantial egg-laying failures necessitated the introduction of individuals from the production population (equivalent to G0) to restore breeding performance. This infusion of successfully reproducing individuals helped re-establish isofamily continuity. However, as a consequence, the mean EBV decreased from G8 to G9, but genetic gain resumed thereafter (G9–G10: +51.2%; G10–G11: +38.2%; G11–G12: +26.6%). Despite this recovery, the magnitude of genetic improvement progressively declined toward the end of the experiment. Selection on the experimental line was therefore discontinued at G12 (Fig. 3).

**Fig. 4:**
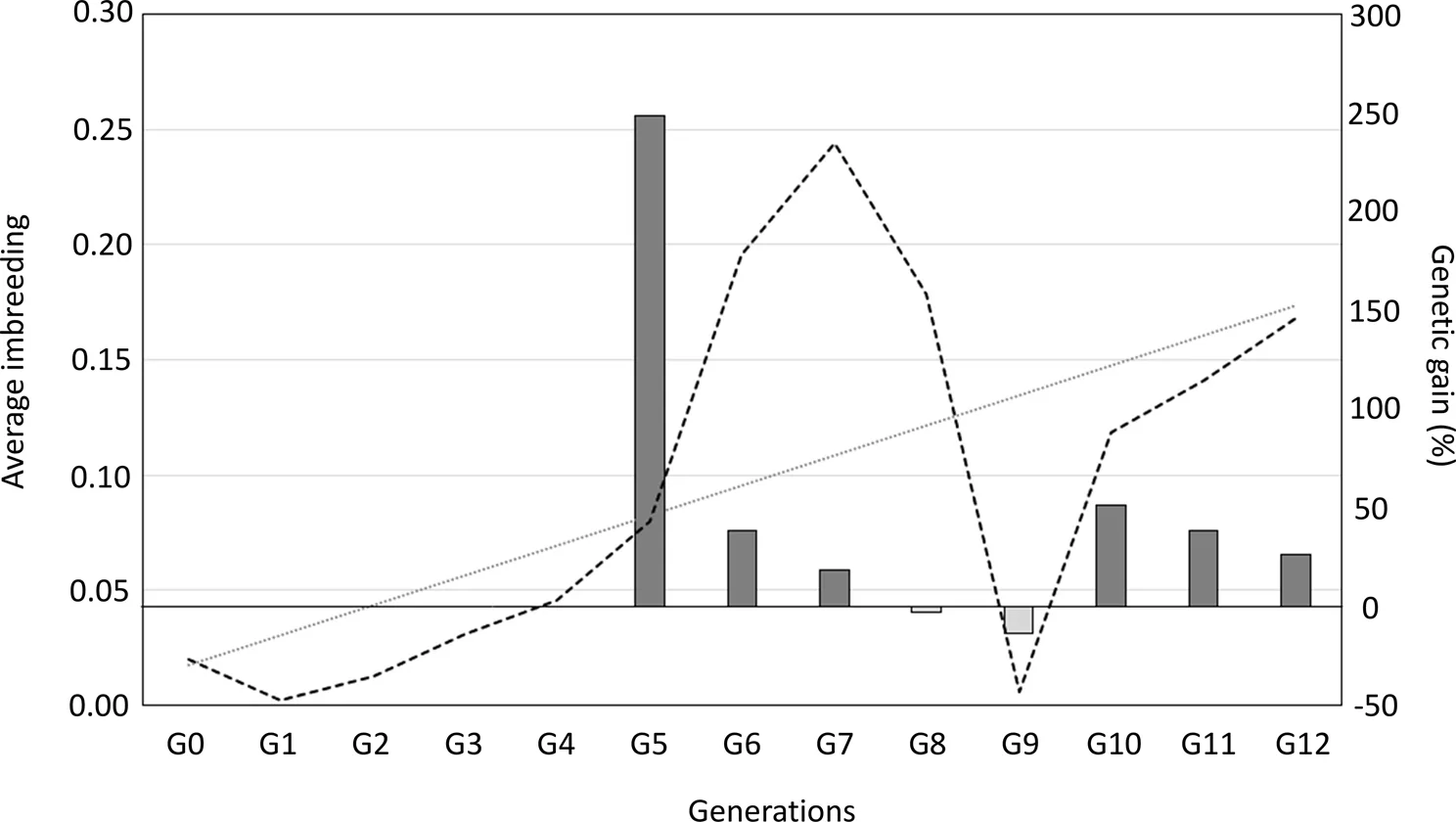
Evolution of genetic gain (dark grey histogram) and average inbreeding (F_mean - dotted curve) through 13 generations of selection on larval body weight (LW2) under a pedigree-based breeding program in *H. illucens*. The light grey line is the linear regression of inbreeding over time. The slope of this curve is 12.69% per year

The genealogical dataset of *H. illucens* revealed a mean inbreeding coefficient of 9.6% across the full population over the thirteen generations monitored, corresponding to an annual increase of 12.7% (Fig. 4). Inbreeding remained low during the initial generations (G0–G4), staying below 5%. From G5 to G8, however, the rate rose sharply, from 7.9 to 24%, reflecting a marked reduction in genetic diversity. This trend was temporarily mitigated in G9, following an admixture event that reintroduced additive genetic variation. Subsequent generations (G10–G12) again displayed a progressive increase, reaching 11.8, 14.11, and 16.8%, respectively (Fig. 4).

### Selection responses and breeding strategies in *H. illucens*

**Fig. 5:**
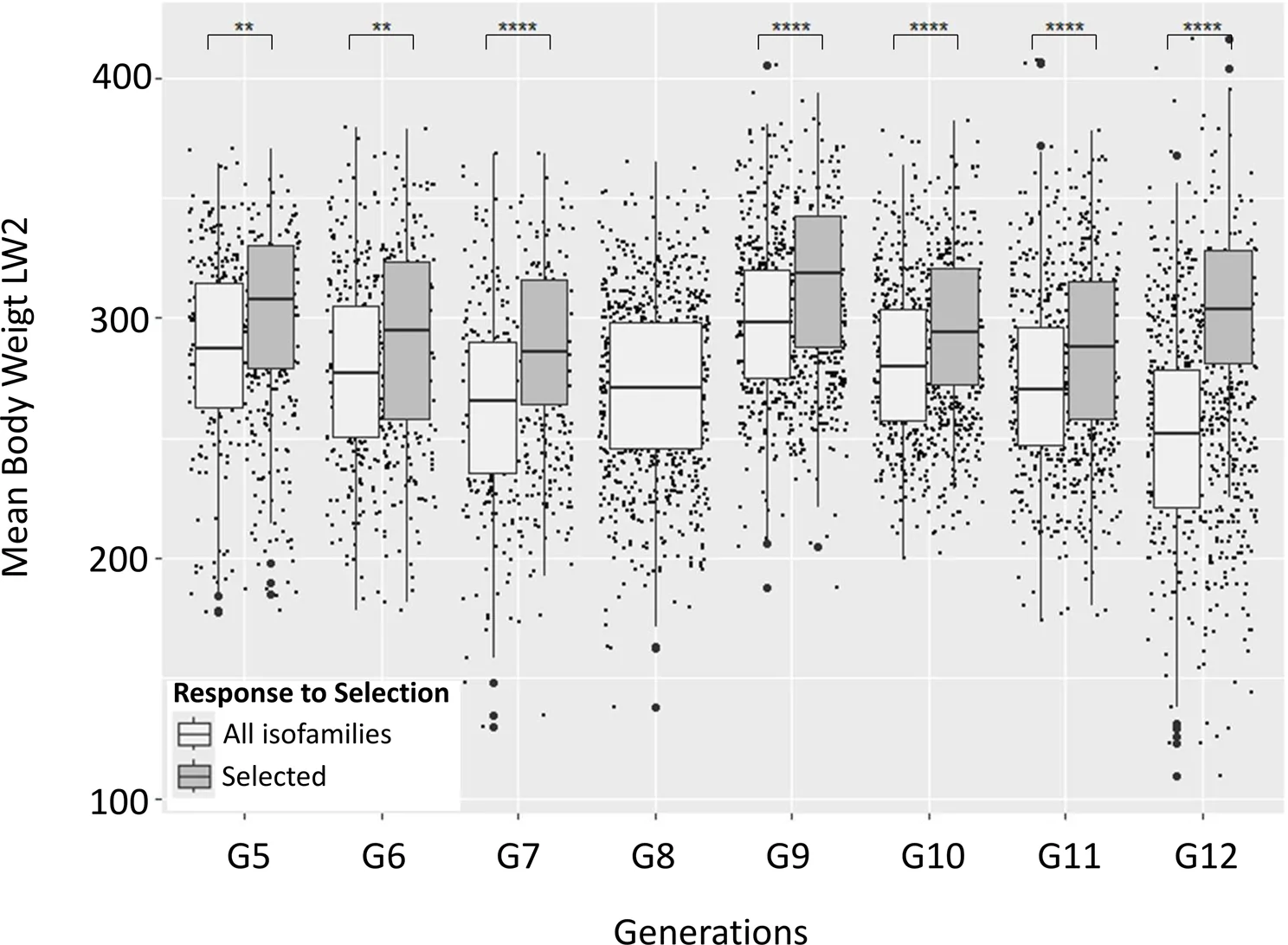
Boxplots comparing mean larval body weight (LW2) between the selected candidates and all isofamilies over generations in *H. illucens*. The boxplots show the median value in the middle notch, and each dot is an individual data point. Asterisks indicate a significant difference between selected and unselected isofamilies (Wilcoxon rank sum test: **0.001 < *p* < 0.01; ***p < 0.001; ns, not significant)

A comparison of LW2 averages revealed consistent and significant differences between selected and unselected isofamilies across the seven consecutive breeding generations evaluated (Fig. 5), confirming the effectiveness of the experimental pedigree-based breeding scheme. The resulting selection intensity, expressed as the selection differential, reached 13.8, 12.4, 25.8, 18.7, 17.2, 15.4, and 56.9 mg for generations G5, G6, G7, G9, G10, G11, and G12, respectively, in the selected lines relative to the unselected controls.

Despite these positive differentials, phenotypic performance for LW2 declined during the early selection phase. The overall population mean decreased from 288 mg in G5 to 280 mg in G6 and 268 mg in G7, indicating a short-term reduction in performance, consistent with the previously described lag in selection response. Following the introduction of additive genetic variance in G8, this trend reversed: the LW2 mean increased to 301 mg in G9, before gradually declining again in subsequent generations, reaching 284, 276, and 262 mg in G10, G11, and G12, respectively.

## Discussion

Animal breeding programs differ substantially across species but are generally structured by requiring reliable pedigree or genomic information, as exemplified in chickens [36, 37], dairy cattle [38, 39], pigs [40], sheep [41, 42] and goats [43]. As in other livestock sectors, genetic improvement of key traits has become an increasing focus for insect production companies, as selective breeding offers a direct route to enhance productivity. In this context, we present a pedigree‑informed selection framework for genetic improvement of the commercially important insect *H. illucens*. Using multivariate REML animal models, we estimated quantitative‑genetic parameters for key growth and reproductive traits and evaluate the feasibility of pedigree‑based selection under industrial rearing. Within this framework, our pilot breeding programme indicates that, with an adequately large and managed selection nucleus, sustained improvement of larval weight (LW2) can be achieved across generations.

### Pedigree accuracy under group mating

The group‑mating design used in this study inherently limits pedigree resolution, as individual parentage cannot be assigned with certainty and all clutches within a cage must be linked to the same parental group. Consequently, the pedigree corresponds to a maternal half‑sib structure with unknown sires [21]. In such systems, progeny necessarily consist of a mixture of full‑ and half‑siblings, which decreases the information content available for estimating additive genetic variance and relatedness coefficients. This structure can also lead to downward bias in inbreeding estimates and reduced accuracy in tracing coancestry across generations [44]. However, the use of large mating groups and systematic rotation of parental cages across generations mitigates the risk of non‑random mating and limits the accumulation of close kinship. While these constraints are inherent to mass‑mating systems and are well recognized in the quantitative‑genetics literature, the pseudo‑pedigree used here follows established practice for analysing group‑mated populations [21, 44]. Accordingly, the resulting structure supports cautious estimation of genetic parameters, albeit with reduced precision compared with controlled pair‑mating designs.

### Genetic parameters and environmental effects on traits of interest

To our knowledge, this pilot‑scale study is one of the first to exploit multi‑generation pedigree information to characterise the genetic architecture of economically important traits in *H. illucens*. Applying an animal‑model approach, we partitioned phenotypic variance and detected indicative additive genetic contributions, alongside shared environmental effects, while acknowledging design‑related limitations.

Studies estimating genetic parameters in insects remain scarce. To date, only a limited number of investigations have reported such estimates in *H. illucens* [15–18, 45], with heritability estimates for growth‑related traits generally ranging from 0.24 to 0.78. These values align well with our estimates. Notably, our study is one of the first to provide a comprehensive set of genetic parameter estimates in *H. illucens*, including genetic correlations among multiple traits and, for the first time, estimates for reproductive traits. Previous studies have relied primarily on selective breeding designs, either mass selection or family selection, typically using only one or a few generations of data. In contrast, our work offers significant added value by using a multi‑generation pedigree to estimate genetic parameters in *H. illucens*, within an isofamily selection framework, where individual data were aggregated at the full‑sib family level. Pedigree depth was known to enhance the accuracy of genetic parameter estimates in classical, individual‑based pedigree designs by improving the separation of genetic and environmental components and by reducing bias due to selection [46, 47]. However, the pseudo‑pedigree structure used here, derived from group mating, does not fully meet these conditions. Consequently, although standard errors were relatively modest, they should be interpreted with caution, as residual uncertainty associated with group mating and environmental aggregation remains.

Previous research on insects that estimated genetic trends from pedigree data shows patterns broadly consistent with our findings. For example, the heritabilities reported by [25] followed similar trajectories, with estimates ranging from 0.10 to 0.68 for production traits and from 0.17 to 0.54 for reproductive traits. Additional studies have also documented heritable variation for key traits in other insect species. These include *Drosophila melanogaster*, with a heritability of 0.58 for adult body weight [48]; the black dump fly *Hydrotaea aenescens,* with heritability estimates between 0.05 and 0.61 for pupal and larval development time [24]) and the house fly *Musca domestica,* showing heritabilities ranging from 0.08 to 0.41 for larval and adult production traits [23].

In our study, common environmental effects were substantial only for larval body weight measured at 7 days post‑hatching (LW1), with an estimated c^2^ of 0.57 ± 0.04. This is consistent with the well‑established sensitivity of early developmental traits to environmental influences, a pattern previously observed in several dipteran species [23, 49]. These results support the view that environmental effects can play a dominant role in shaping early‑life phenotypes in insects, while later traits tend to be increasingly governed by additive genetic variation.

Among the production traits, body weight at 14 days post‑hatching under limiting feeding conditions (LW2R) showed a higher heritability (h^2^ = 0.58 ± 0.03) than the same trait measured under non‑limiting conditions (LW2; h^2^ = 0.13 ± 0.04). This pattern was confirmed in an independent experimental line (unpublished data) monitored over four generations. Such findings have practical relevance for industrial entomoculture, as they suggest that selecting for performance under rationed conditions could allow the improvement of a key production trait at reduced cost. Beyond the economic implications, these results also raise questions regarding the role of common environmental effects in *H. illucens* breeding. Our analyses show that the phenotypic variance of LW2 depends strongly on the local rearing environment (e.g. rationed vs. non‑rationed feeding), which likely contributes to the differences in genetic variance components estimated for LW2 and LW2R. Similar environment‑dependent changes in phenotypic and genetic variance have been documented in multiple *H. illucens* strains reared under different diets [50, 51], where significant strain‑by‑diet interactions were observed for growth‑related traits. Such discrepancies may also reflect genotype‑by‑environment interactions detected for LW2 in our internal research, further supporting the hypothesis that environmental context modulates both the expression and the genetic architecture of this trait.

Regarding genetic correlations, the results must be interpreted cautiously due to the relatively large associated standard errors. Nevertheless, the estimates suggest favourable positive genetic correlations among production traits (LW1, LW2, LW2R, PERIM) and among reproductive traits (OOCY, THOR). Results consistent with these estimates have been reported in empirical selection studies on the yellow mealworm *Tenebrio molitor*, where selection for increased body size led concurrently to enhanced fecundity [52, 53]. Such positive genetic correlations provide a promising basis for developing multi‑trait breeding objectives that simultaneously improve both production and reproductive performance.

### Body weight (LW2) selection: potential and inbreeding risks

Using pedigree information for genetic selection on body weight in *H. illucens* is likely to be valuable only when integrated into a long‑term multi‑trait breeding program, in which the balance between implementation cost and expected genetic gain is carefully optimised [54]. A recent long‑term selection study demonstrated that substantial increases in larval body weight can be achieved over 16 generations using mass selection alone [14]. However, such schemes do not allow control of inbreeding accumulation and therefore pose a considerable risk of inbreeding depression and reduced population robustness. In contrast, BLUP has become the standard methodology for estimating breeding values in livestock breeding programs [55, 56], as it efficiently incorporates information from related individuals and maximises the use of available data in comparisons across progeny groups. In the context of our LW2 experimental pilot‑scale selection program in *H. illucens*, we used a single‑trait BLUP animal model to predict breeding values for LW2. This provides a workable basis for selection under industrial rearing, enabling incremental gains in accuracy and ongoing inbreeding surveillance, while acknowledging uncertainties associated with pseudo-pedigree and environmental aggregation.

The average larval body weight of *H. illucens* exhibited substantial fluctuations during the one-year experimental period, encompassing eight generations of continuous selection for increased isofamily mean body weight based on EBVs for LW2. During the initial management phase (G0–G4), random isofamily crosses were performed to stabilise egg production prior to initiating directional selection. Accordingly, the sustained increase in EBVs observed from G5 to G7 (Fig. 3) reflects the potential effect of selection. However, the apparent reduction in additive genetic variance and the plateauing or even decline of genetic gain across these generations likely stem from the concurrent and substantial increase in inbreeding, which reached approximately 25% in G7 (Fig. 3). Under the current selection implementation, the calculated inbreeding values should be viewed as approximate indicators of kinship rather than exact inbreeding coefficients because of pedigree imprecision. Within this context, the observed performance declines over generations are consistent with inbreeding accumulation, in line with the observed reductions in oviposition and hatching rates. A marked shift occurred when G8 hybrids were crossed to individuals from the original parental population, with immediate increases in additive variance and a notable decrease in kinship, compatible with an admixture‑driven restoration of diversity.

Relationships between genetic selection and inbreeding have already been reported in *H. illucens*. For example [13], showed a collapse of a population after only five generations as a consequence of reduced genetic diversity. Similar detrimental effects of inbreeding have been described in other insects, notably in *Drosophila ampelophila*, where inbreeding negatively affected fecundity [57]. Although this relationship is often associated with reduced performance in the selected traits [58], exceptions exist. In the flour beetle *Tribolium castaneum*, for instance, inbreeding did not impair resistance to parasitic nematodes [59]. Likewise, in the bumblebee *Bombus terrestris*, comparisons between the offspring of sib-mated colonies and those of outbred colonies revealed no measurable effects of inbreeding on immune response or body size in either workers or haploid males under different rearing conditions [60]. Our results suggest that EBV-based selection for body weight was associated with an increase in genetic merit across generations, but concurrently accelerated the accumulation of inbreeding. This pattern suggests that selection intensity, combined with limited population size, contributed to the observed rise in inbreeding across the generations studied. Since inbreeding is defined as zero in the base population, the accumulation of inbreeding observed in subsequent generations can be attributed to the selection and mating decisions applied within the breeding program. We hypothesise that the relatively small effective size of the initial selection nucleus accelerated this process by limiting the available genetic diversity and increasing the probability of matings among related individuals.

### Future directions for pedigree-based genetic improvement in *H. illucens*

The reproductive cycle of *H. illucens*, although rapid and central to breeding management, presents several zootechnical constraints that complicate its integration into structured breeding programs. In our proposed breeding scheme, initially inspired by a maternal isofamily-based design developed for *Drosophila melanogaster* [30], we assumed that each collected clutch, considered as a full-sib isofamily, was produced by a single male–female pair. At the time of our study (2019), no data were available regarding polyandry in *H. illucens*. Since then, several studies have demonstrated that polyandry is in fact common in this species, with females frequently remating and producing clutches fertilised by multiple males [61, 62]. These findings directly challenge the assumption of strict full-sib family structure. Thus, while using single-female oviposition as a basis for constructing a family-structured breeding design remains a practical operational choice, it should not be interpreted as guaranteeing full sibship. Complementary internal observations of mating behaviour, together with a parentage-assignment study conducted using recently developed genomic tools for *H. illucens* [63], indicate a pedigree misassignment rate of 10 to 20% within our system. Such pedigree uncertainty likely affected the standard errors precision of estimates of genetic parameters, although its exact impact cannot be quantified. Importantly, true selection gains for LW2 may have been greater than those observed if pedigree assignment had been error-free. Given the group-based rearing and reproduction system inherent to *H. illucens*, and the compact yet spatially constrained structure of clutches, achieving 100% accuracy in pedigree recording is challenging. Although double oviposition within a single cavity appears unlikely based on our experimental observations, we acknowledge that it cannot be categorically ruled out. Consequently, some level of pedigree imprecision is unavoidable in our family-based breeding scheme for this species, and this should be explicitly considered when replicating this breeding selection program and interpreting genetic parameter estimates.

Despite these limitations, it is important to note that pedigree inaccuracies are not unique to insect breeding. In other livestock sectors, most notably dairy cattle, pedigrees used for genetic evaluation routinely contain errors [64]. Reported pedigree error rates across global dairy cattle populations range from 5 to 15%, and in some cases exceed 20% [65]. Yet, these inaccuracies have not prevented effective and sustained genetic improvement in cattle productivity, illustrating that moderate levels of pedigree uncertainty can be compatible with highly successful breeding programs. In the context of black soldier fly breeding, our results demonstrate substantial potential for implementing genetic selection across all traits studied, particularly for larval weight under rationed conditions, which showed high heritability.

According to a recent study comparing phenotypic, pedigree‑based, and family‑based selection in insects using stochastic simulation [66], the most effective strategy for *H. illucens* breeders aiming to apply pedigree‑based selection is to significantly increase the number of families contributing to the breeding nucleus from approximately 60 to 200 or even more. Expanding the number of families reduces inbreeding risk, increases effective population size, and ultimately improves the reliability and long‑term sustainability of selection schemes in *H. illucens.*

## Conclusions

Our study is one of the first to implement and report the outcomes of a family‑based pedigree selection program in *H. illucens*. The estimated genetic parameters including heritabilities, common environmental variance components, and genetic correlations, were broadly consistent with estimates reported in the limited existing literature. Selection on EBVs for larval body weight (LW2) resulted in temporal patterns that are consistent with a genetic response under industrial breeding conditions, although environmental influences cannot be fully excluded in the absence of a contemporaneous control line. These trends persisted until increasing levels of inbreeding were associated with reduced growth performance. Overall, the results indicate that LW2 harbours substantial potential for genetic improvement, while also highlighting the necessity of managing inbreeding risk through an appropriately sized effective nucleus and careful pedigree management. More broadly, as in other livestock sectors, sustainable genetic improvement ultimately requires the consideration of multiple traits simultaneously, typically through the use of selection index. Future pedigree‑based breeding programs for *H. illucens* should therefore move beyond single‑trait selection and focus on multi‑trait breeding objectives that integrate production, reproductive, and robustness traits to ensure long‑term genetic progress and population stability.

## Data Availability

The data that support the findings of this study are available from the corresponding author upon reasonable request.
